# Electrical Mechanism

**Published:** 1887-11

**Authors:** 


### ELECTRICAL MECHANISM.

We extract the following from an address delivered at a recent annual meeting of the American Institute of Electrical Engineers by Mr. T. C. Martin.

"The electric statistics of to-day are nothing short of colossal. To go no farther than our own country, we find an investment and capitalization in simply the leading electrical industries—telegraphy, telephone, electric lighting and electric power—of a sum of at least $350,000,000. There are in America at least 700,000 miles of telegraph wire. Last year 320,000,000 telephonic conversations were held. Over 150,000 arc lamps, and incandescents verging on 1,000,000 now instruct the public eye, that the electric light is the light before which all others must "pale their ineffectual fires." At least 10,000 electric motors are employed in the most varied industries, and while the electric railways here are carrying passengers at the rate of 3,500,000 annually, the extraordinary increase now going on in that branch, promises that the figures will be doubled within the year with at least from 50 to 75 electric roads in operation. When to these figures we add the thousands of dynamos and vats used in electro-plating, the countless electrical appliances used in medicine, surgery and the miscellaneous arts, and the endless variety of electric apparatus for convenience, safety, protection, and comfort in the house, office, train or hotel, we arrive at sum totals dealing with which a statistician needs all his ability to mass and classify. It is no exaggeration to say, moreover, that in the electric industries hundreds of thousands of our most intelligent citizens of both sexes, find remunerative and agreeable occupation ; nor is it exaggeration to affirm that at a time when all great departments of manufacture must depend for success on the degree of technical culture and skill displayed in them, none rests on a more strictly scientific basis than ours."

